# Both PAX4 and MAFA Are Expressed in a Substantial Proportion of Normal Human Pancreatic Alpha Cells and Deregulated in Patients with Type 2 Diabetes

**DOI:** 10.1371/journal.pone.0072194

**Published:** 2013-08-27

**Authors:** Rémy Bonnavion, Rami Jaafar, Julie Kerr-Conte, Fouzia Assade, Esther van Stralen, Emmanuelle Leteurtre, Célio Pouponnot, Sofia Gargani, François Pattou, Philippe Bertolino, Martine Cordier-Bussat, Jieli Lu, Chang Xian Zhang

**Affiliations:** 1 Inserm U1052, Lyon, France; 2 CNRS UMR5286, Lyon, France; 3 Université de Lyon, Lyon, France; 4 The E-Institute of Shanghai, Sino-French Life Science and Genomic Center, Ruijin Hospital, Shanghai, China; 5 Shanghai Clinical Center for Endocrine and Metabolic Diseases, Ruijin Hospital, Shanghai Jiao-Tong University, Shanghai, China; 6 Université Lille Nord de France/INSERM U859 Biotherapies of Diabetes, Faculty of Medicine, Lille, France; 7 Department of Pathology, Centre Hospitalier Régional et Universitaire de Lille, Lille, France; 8 Institut Curie, CNRS UMR3347, INSERM U1021, Orsay, France; 9 Hubrecht Institute, Royal Netherlands Academy of Arts and Sciences & University Medical Centre Utrecht, Utrecht, The Netherlands; Peking Union Medical College Hospital, Peking Union Medical College, Chinese Academy of Medical Sciences, China

## Abstract

Pax4 and MafA (v-maf musculoaponeurotic fibrosarcoma oncogene homolog A) are two transcription factors crucial for normal functions of islet beta cells in the mouse. Intriguingly, recent studies indicate the existence of notable difference between human and rodent islet in terms of gene expression and functions. To better understand the biological role of human PAX4 and MAFA, we investigated their expression in normal and diseased human islets, using validated antibodies. PAX4 was detected in 43.0±5.0% and 39.1±4.0% of normal human alpha and beta cells respectively. We found that MAFA, detected in 88.3±6.3% insulin^+^cells as in the mouse, turned out to be also expressed in 61.2±6.4% of human glucagons^+^ cells with less intensity than in insulin^+^ cells, whereas MAFB expression was found not only in the majority of glucagon^+^ cells (67.2±7.6%), but also in 53.6±10.5% of human insulin^+^ cells. Interestingly, MAFA nuclear expression in both alpha and beta cells, and the percentage of alpha cells expressing PAX4 were found altered in a substantial proportion of patients with type 2 diabetes. Both MAFA and PAX4 display, therefore, a distinct expression pattern in human islet cells, suggesting more potential plasticity of human islets as compared with rodent islets.

## Introduction

The development and functions of different types of islet cells are controlled, to a large extent, by essential cell-lineage-specific transcription factors. Among them, the transcriptional regulators Pax4 and Maf are of crucial importance for both cell differentiation and normal functions of islet beta cells in the mouse [1], [2]. Pax4 is mandatory for the development and maturation of mouse beta cells [1]. The evidence from the recent studies suggest that PAX4 is also crucial for mature beta cell expansion and survival, and that PAX4 mutations or polymorphisms are associated with both type 1 and type 2 diabetes [3]. Importantly, Pax4 overexpression in mouse pancreatic progenitors resulted in beta cell conversion [4]. MafB is involved in embryonic development of both mouse pancreatic alpha and beta cells [5], [6], whereas MafA participates mainly in the maturation of mouse beta cells [2]. During adult life in the mouse, MafB is specifically expressed in alpha cells [6] and MafA in beta cells [2]. Interestingly, we and others have recently demonstrated that MafB expression can be reactivated in mouse beta cells undergoing adaptive proliferation [7], [8] and during tumorigenesis [7], indicating that the dynamic modulation of Maf expression could be involved in the control of beta cell proliferation and their endocrine functions. Based largely on the knowledge acquired from the above mentioned studies in the mouse, several strategies to cure diabetes are aimed at replenishing the pool of beta cells, by overexpressing beta cell specific transcription factors, including Pax4, MafA and Pdx1 in different cell-types.

Although the expression pattern and biological functions of these islet transcription factors have been substantially explored in rodent models and cell lines, little was known in human islets until recently. *PAX4* mRNA was undetectable [9] and PAX4 protein expression has been barely studied in normal adult human islets, mainly because of the lack of specific tools. Intriguingly, Dorrell *et al*. recently reported that MAFB was detected at equal levels in FACS purified adult human alpha and beta cells by RT-qPCR analysis, suggesting that MAFB expression in human islets may be different from that in mouse [9]. Consistent with this work, Dai *et al*. subsequently reported MAFB protein expression by immunohistochemistry in a rather small proportion of beta cells in normal adult human islets [10]. Considering the marked difference reported between MAFB expression in human and murine islets, the goal of this study was to find out detailed protein expression patterns of PAX4 and MAFA in human adult alpha and beta cells.

## Materials and Methods

### Ethics statement

Human paraffin embedded pancreatic tissues from the collection (CHRU Lille, France) conserved for traceability in the context of our clinical islet transplant program [11] were obtained from healthy subjects (n = 9), Type 2 Diabetics (n = 7), and non-diabetic subjects with obesity (n = 4, Table 1). Verbal consents from the donor were obtained for use of the samples in research. The study was conducted in accordance with the guidelines in the Declaration of Helsinki. Ethical review board (« Comité d'Ethique du Centre Hospitialier Régional et Universitaire de Lille ») approved the use of human pancreatic cells in vitro, and their associated paraffin sections. Authorization to access research grade pancreases is granted by Agence de la BioMedecine, France. The Agence de la BioMedecine has standardized procedures for Coordinators to assure that the informed consent procedure is identical throughout France.

**Table 1:** Human patient samples.

Patient#	Sex	Age (year)	BMI (kg/m^2^)	HbA1c (%)	Clinical features
**1**	M	64	39.2	10.3%	T2D
**2**	M	58	32.8	7.0%	T2D
**3**	M	62	32.7	7.4%	T2D
**4**	M	49	29.9	7.7%	T2D
**5**	M	57	36.5	8.2%	T2D
**6**	F	55	22.4	7.7%	T2D
**7**	M	59	37.7	7.0%	T2D
**Mean ± S.E.M T2D**	**–**	**57.7±1.8**	**33.0±2.2**	**7.9±0.4%**	**–**
**8**	M	66	36.9	5.3%	Control obese
**9**	F	50	33.3	6.2%	Control obese
**10**	F	46	33	5.7%	Control obese
**11**	M	55	39	5.8%	Contol obese
**Mean ± S.E.M Control obese**	**–**	**54.3±4.3**	**35.6±1.5**	**5.8±0.2%**	**–**
**12**	M	18	21.6	5.6%	Control
**13**	M	17	23.7	5.6%	Control
**14**	M	18	23.3	ND	Control
**15**	M	41	22.2	5.5%	Control
**16**	M	36	24.2	ND	Control
**17**	M	43	22.5	5.6%	Control
**18**	F	47	25.7	ND	Control
**19**	F	47	20.8	5.7%	Control
**20**	H	48	24,7	ND	Control
**Mean ± S.E.M Control**	**–**	**35.0±4.5**	**23.2±0.5**	**–**	**–**

BMI: Body Mass Index; HbA1c: Glycated Hemoglobin; ND: Not Determined.

### Cell transfection and western-blotting

αTC1-9 were transfected with the empty vector pCI-neo, pCI-mPax4 or pCI-hPAX4 expressing mouse or human PAX4 respectively and were analysed as previously described [7]. MEF cells were transfected with pcDNA-MAFA and pcDNA-MAFB expressing human MAFA and MAFB, respectively, and analysed as previously described [7].

### Pax4 vectors cloning

Mouse Pax4 expressing vector was constructed by subcloning of a PCR fragment obtained from βTC3-cell line cDNA into the pCI-neo mammalian expression vector and was verified by sequencing. The following primers containing SalI and NotI restriction sites were used: 5′-ATAAGTCGACATGCAGCAGGACGGACTCAGC-3′ and 5′ATAAGCGGCCGCTTATGGCCAGTTTGAGCAATG-3′. Human PAX4 expressing vector was constructed by subcloning of a PCR fragment obtained from a human insulinoma cDNA into the pCI-neo mammalian expression vector and was verified by sequencing. The following primers containing SalI and NotI restriction sites were used: 5′-ATAAGTCGACATGCATCAGGACGGGATCAGC-3′ and 5′- ATAAGCGGCCGCTTAAGGCCAGTGTGAGAAGTG -3′.

### Antibodies used

The anti-MAFA antibody (ab26405, Abcam) and a panel of anti-MAFB antibodies were subjected to different tests to assess their specificity toward MAFA and MAFB proteins both in mouse and in human tissues. The anti-MAFA (ab26405, Abcam) and the noncommercial anti-MAFB2 (mouse monoclonal antibodies, clone 1F4 [12]) antibodies were validated and used for the current study. Anti-glucagon (G2654, Sigma), the anti-insulin (A056401, DAKO), the anti-PDX1 (ab47383, Abcam) and anti-ARX (noncommercial, [13]), mMafB1 (anti mouse MafB, IHC-00351, Bethyl), and hMAFB3 (Anti human MAFB, HPA005653, Sigma) were also used in the study.

### Immunofluoresence analyses

Briefly, serial sections (4 μm) mounted on glass slides were de-waxed and rehydrated through a series of ethanol dilutions then distilled H_2_O. Heat-induced epitope retrieval was performed by immersion in Antigen Unmasking Solution (H-3300, Vector) in a microwave oven. After blocking with antibody diluent (DAKO) sections were incubated with a primary antibody, and treated with Cy-3 or Cy-5 Tyramide amplification kit (PerkinElmer) according to the manufacturer′s instructions or incubated with appropriate Alexa488–555–or–647 coupled secondary antibodies (Life Technologies). For PAX4 detection in human pancreas, heat-induced epitope retrieval was not performed but sections were incubated in PBS-Triton X100 0.2% for 5 min at room-temperature. However this prevented us from performing ARX and PAX4 co-expression because ARX could not be detected with this protocol. All nuclear co-localization experiments were performed and controlled to ensure that no non-specific crossreaction occurred. Images were captured and analyzed on a TCS-SP5 confocal microscope (Leica-Microsystems) or a Zeiss 780 confocal microscope. Cell counting was manually performed with ImageJ cell counter module (U. S. National Institutes of Health, Bethesda, Maryland, USA) on multiple channel pictures. Graphs and results of counting are represented as mean ± S.E.M.

## Results

### Endogenous PAX4 nuclear expression can be detected both in human pancreatic alpha and beta cells

**Figure 1:**
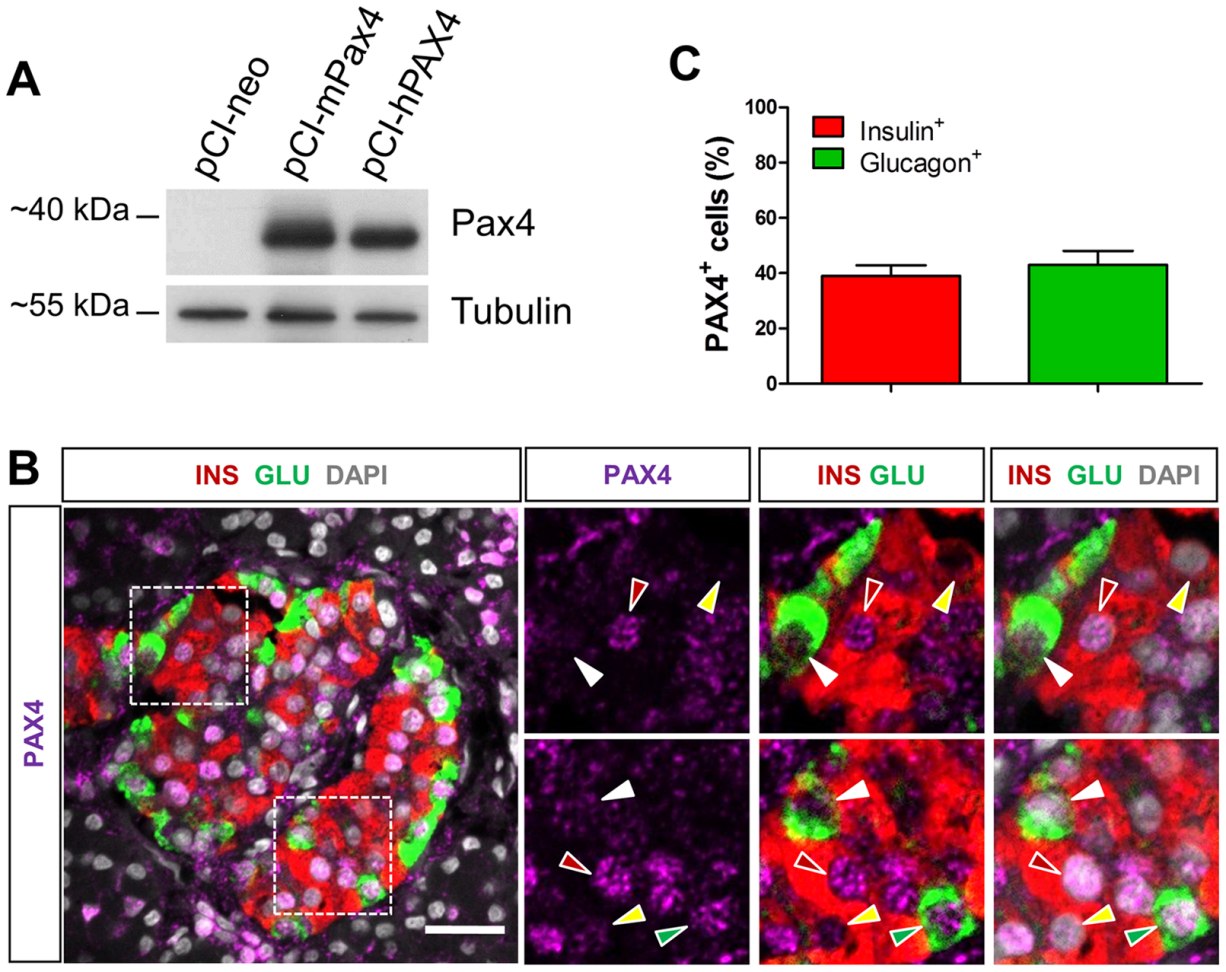
PAX4 is detected in both human pancreatic alpha and beta cells. (A) Detection of ectopically expressed mouse and human PAX4 by western blotting. The capacity of the anti-Pax4 antibody to recognize human PAX4 was assessed using αTC1-9 cells transfected respectively with the pCI-neo empty vector or constructs expressing mouse Pax4 (mPax4) or human PAX4 (hPAX4) protein by western-blotting analysis. Protein extracts from transfected cells were used for the detection, using antibody against Pax4. (B) Representative images of triple IF staining with antibodies against PAX4 together with glucagon and insulin. Right panels are the amplified view of the insets in the left panel. Red arrowheads, PAX4^+^ INS^+^ cells. Yellow arrowheads, PAX4^−^INS^+^ cells, Green arrowheads, PAX4^+^GLU^+^. White arrowheads, PAX4^−^GLU^+^ cells. (C) Percentages of insulin^+^ cells and glucagon^+^ cells expressing PAX4 represented as the averaged counting results ±S.E.M from n = 5 control individuals (A total of 164 GLU^+^ cells and 904 INS^+^ cells were analyzed). Scale bars  = 25 µm.

First, we sought to validate the capacity of a widely used anti-Pax4 antibody [4], [14], [15] to recognize human PAX4 (Figure 1A) expressed in αTC1-9 cells, since the antibody has not been reportedly used in human pancreatic tissues. As illustrated in Figure 1A, the antibody recognized properly ectopically expressed human PAX4 and its mouse counterpart. Using triple IF staining of PAX4, glucagon, and insulin, we found that surprisingly, PAX4 was expressed with an equivalent intensity in 43.0±5.0% and 39.1±4.0% of normal human alpha and beta cells respectively (Figure 1B, C). The relative expression intensity was much less in human islets than in mouse islet beta cells (data not shown). Importantly, this unexpected result is consistent with reported weak *PAX4* mRNA expression found in both human adult alpha and beta cells [9].

### Expression patterns of MAFA and MAFB are largely distinct in human endocrine pancreas than those in the mouse

**Figure 2:**
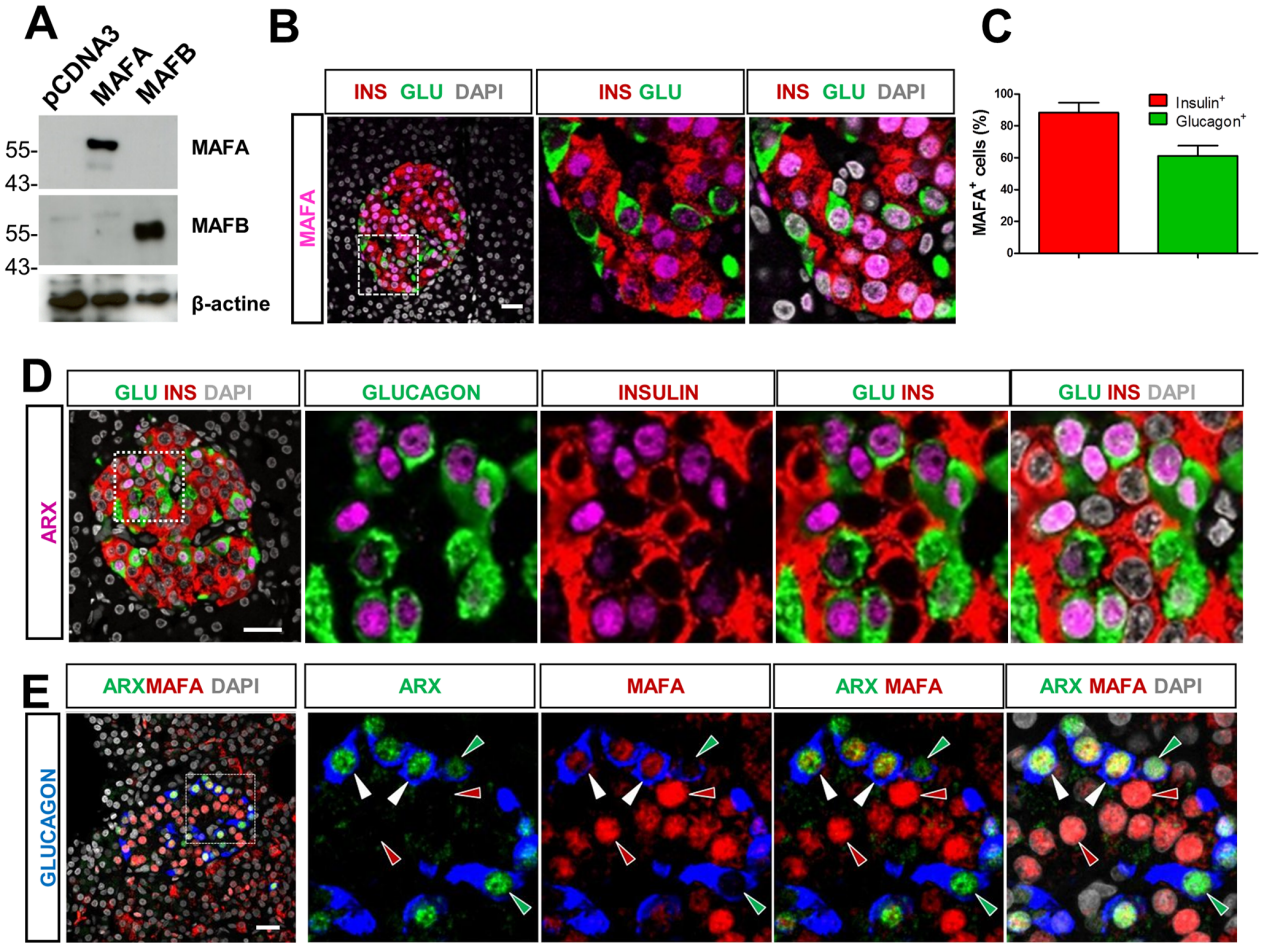
MAFA is detected in both human pancreatic alpha and beta cells. (A) Detection of ectopically expressed human MAFA and MAFB by western blotting. The specificity of selected antibodies against human MAFA and MAFB were evaluated using mouse embryonic fibroblasts (MEF), which do not express endogenous murine MafA and MafB proteins, transfected respectively with constructs expressing human MAFA or MAFB protein by western-blotting analysis. Protein extracts from MEF transfected with empty pcDNA and pcDNA expressing respectively human MAFA and MAFB were used for the detection, using antibodies against MAFA (Abcam) or MAFB (anti hMAFB2, mouse monoclonal antibodies, clone 1F4). Note that the anti-MAFA antibody and the noncommercial anti-hMAFB2 reacted specifically without cross-reaction, whereas the other tested commercially available anti-MAFB antibodies failed (data not shown) (B) Triple immunofluorescent (IF) staining showing MAFA expression in human islets from healthy donors. (C) The percentages of cells expressing MAFA were 88.3±6.3% MAFA^+^ beta cells and 61.2±6.4% MAFA^+^ alpha cells. Results are the averaged expression ±S.E.M of counting results from n = 4 control individuals (1058 INS^+^ cells and 345 GLU^+^ cells were counted in total). (D) ARX expression was detected in human pancreatic alpha cells but not human pancreatic beta cells. Representative images of triple IF-staining showing ARX expression in human islets from healthy donors. ARX was detected only in nuclei of alpha cells. (E) Co-localisation of MAFA with ARX in human islets. Right panels are the amplified view of the inset in the left panel. Red arrowheads, MAFA^+^ARX^−^GLU^−^cells. Green arrowheads, MAFA^−^ARX^+^GLU^+^. White arrowheads, MAFA^+^ARX^+^GLU^+^ cells. Scale bar  = 25 µM.

**Figure 3:**
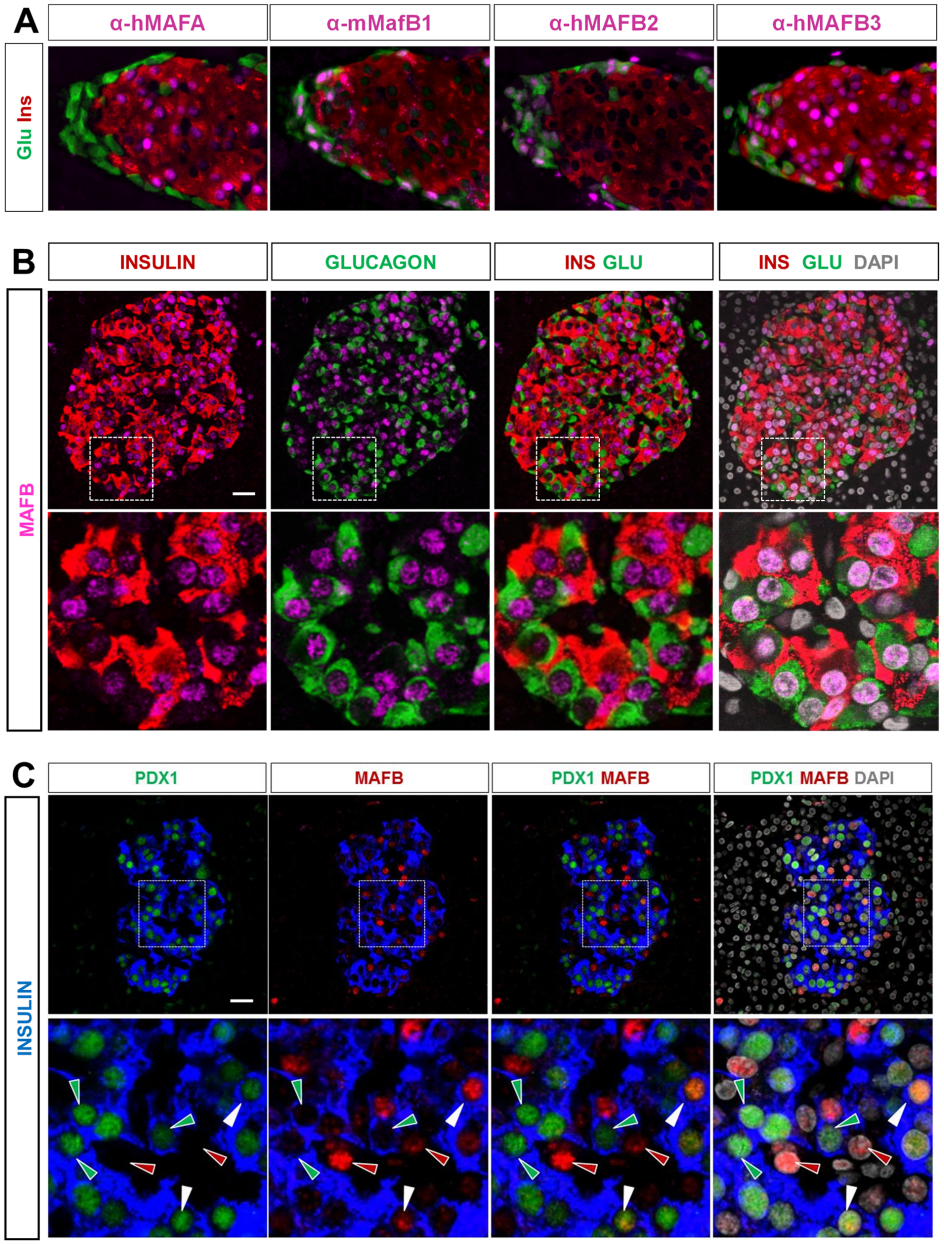
MAFB is expressed in both human alpha and beta cells. (A) Different antibodies were tested for their specificity toward mouse MafB by triple immunofluorescent (IF) staining of mouse pancreatic sections. Representative images of triple IF staining with anti-glucagon, insulin, and hMAFA, mMafB1, hMAFB2 and hMAFB3 antibodies. Note that hMAFB3 did not show an alpha cell specific pattern as did mMafB1 and hMAFB2 antibodies. (B) MAFB expression was detected in both human pancreatic alpha and beta cells. Representative images of triple IF-staining showing MAFB expression in human islets from healthy individuals. The percentages of positive cells for MAFB in glucagon^+^ (green) and insulin^+^ (red) cells were 67.2±7.6% MAFB^+^ alpha cells and 53.6±10.5% MAFB^+^ beta cells. Results are the averaged expression ± S.E.M of counting results from n = 4 control individuals (463 GLU^+^ cells and 797 INS^+^ cells were counted in total). Scale bar  = 25 µM. (C) MAFB expression in insulin^+^ PDX1^+^ cells. The percentage of double insulin^+^ PDX1^+^ cells positive for MAFB was 30.2±5.8% (858 INS^+^ PDX1^+^ counted cells in total). The above percentages of expression are the averaged counting results ±S.E.M from n =  3 control individuals. Scale bar  = 25 µM.

Then, to better investigate endogenous expression of MAFA and MAFB in human islets, we validated the specificity of anti-MAFA and MAFB antibodies used in the current study. We found that the anti-MAFA antibody (Abcam) and the non-commercial anti-hMAFB2 (mouse monoclonal antibodies, clone 1F4 [12]) reacted specifically without cross-reaction both to their corresponding human counterpart ectopically expressed in mouse embryonic fibroblasts (Figure 2A) and their corresponding protein in mouse pancreas (Figure 3A). These antibodies were then further validated for the detection of endogenous human MAFA and MAFB expression in fixed human pancreatic sections by IHC (data not shown).

As expected, the validated antibodies recognized human MAFA in the nucleus of most insulin^+^ cells (88.3±6.3%) on sections of adult human pancreas from healthy individuals (Figure 2B and C). Surprisingly, as opposed to what was observed in mouse alpha cells, MAFA was also detected in more than half of human glucagon^+^ cells (61.2±6.4%, Figure 2B and C). MAFA expression in alpha cells was confirmed by colocalisation with an alpha cell-specific transcription factor, ARX (Figure 2D and 2E). In parallel, using an antibody recognizing specifically both mouse and human MAFB (Figure 2A and 3A), we found that 67.2±7.6% of human glucagon^+^ cells displayed an overt MAFB staining, yet MAFB was also detected in a large number of insulin^+^ cells (53.6±10.5%, Figure 3B), and double insulin^+^ PDX1^+^ cells (30.2±5.8%, Figure 3C), similar to what was reported in a recent study but to a larger extent [16].

### MAFA and PAX4 expression is found deregulated in patients with type 2 diabetes

**Figure 4:**
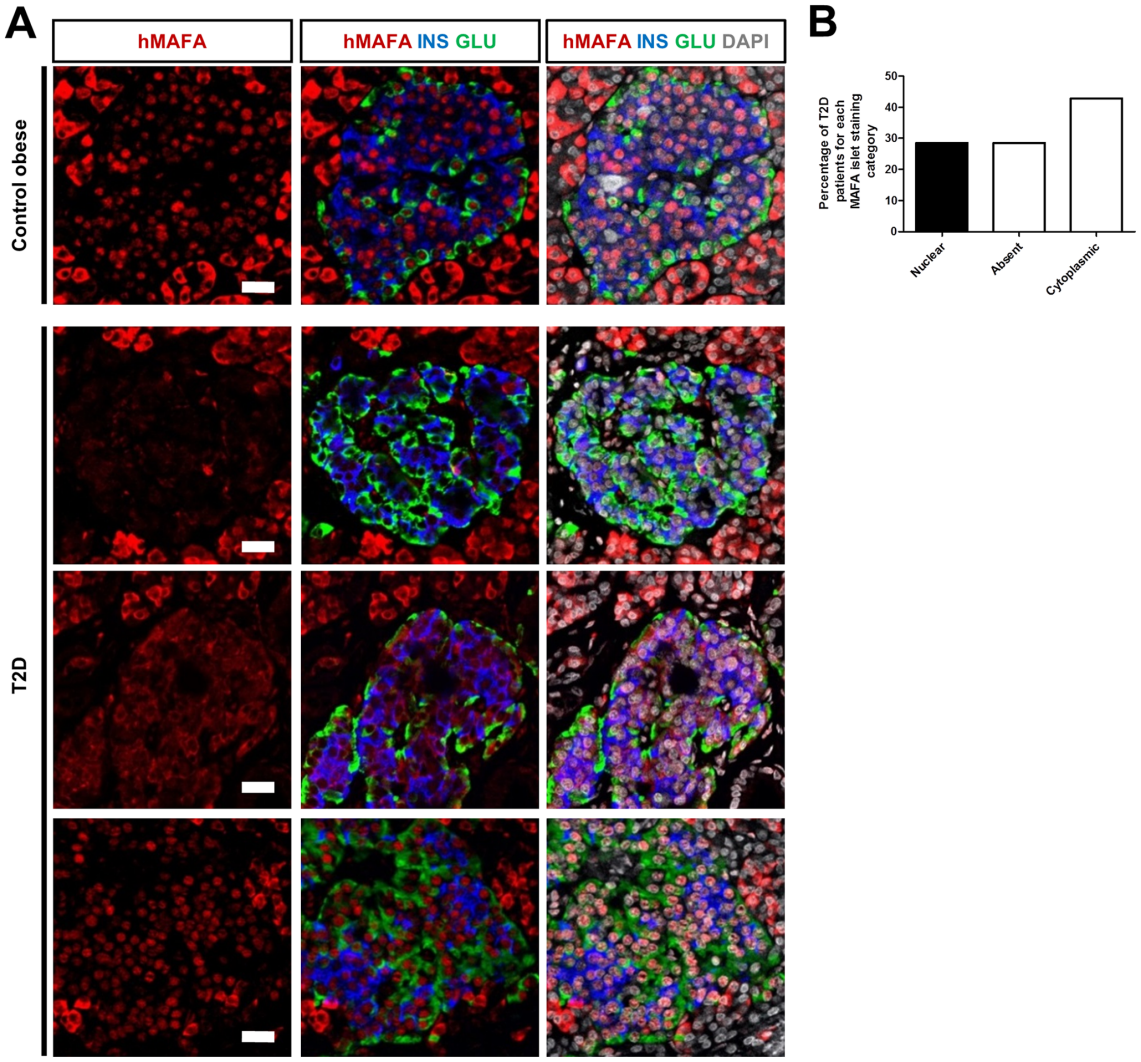
MAFA expression in islets from subjects with obesity or T2D diabetes. (A) Representative images of triple IF staining of MAFA together with insulin and glucagon in subjects with obesity and T2D patients showing a MAFA-negative (upper panels), a MAFA-cytoplasmic (middle) and a nuclear MAFA staining (lower panels) were respectively shown. (B) Percentage of T2D patients displaying each category of prominent MAFA expression in islets. Scale bars  = 25 µm.

**Figure 5:**
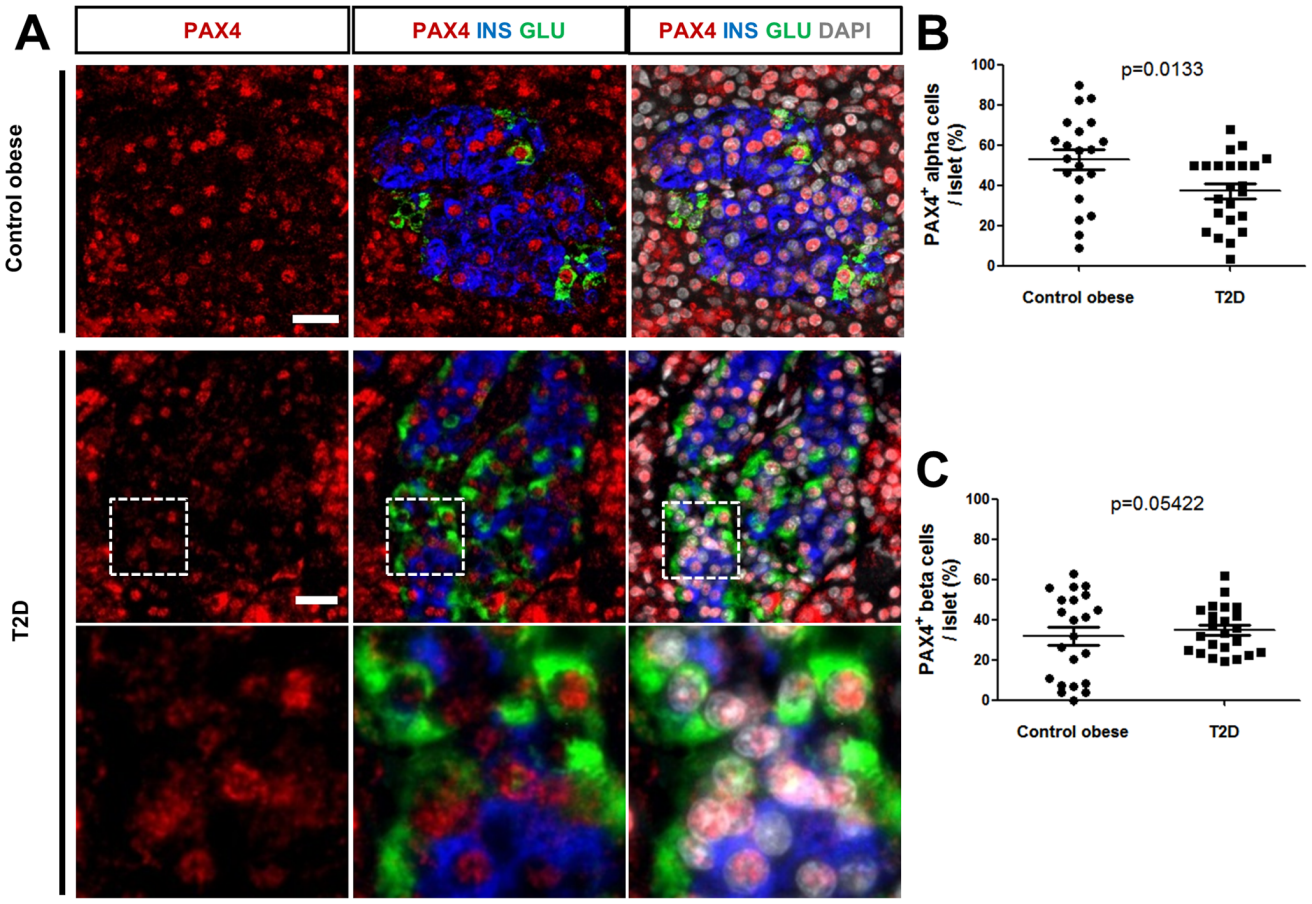
PAX4 expression in islets from subjects with obesity or T2D diabetes. (A) Representative triple IF of PAX4 together with insulin and glucagon showing PAX4-positive islet from control subject with obesity and T2D patients. The lower panel represents a magnified view of the insets in the upper panel. Scale bars  = 25 µm. (B) Overall distribution of PAX4^+^GLU^+^ cells per islet from control subjects with obesity and T2D patients (52.8±4.8% with n =  total of 21 islets from 4 control subjects with obesity vs 37.2±3.7% with n =  total of 23 islets from 5 T2D patients, p = 0.0133 using unpaired two-tailed Student's *t* test. (C) Overall distribution of PAX4^+^INS^+^ cells per islet from control subjects with obesity and T2D patients (31.7±4.5%, n =  total of 22 islets from 4 control subjects with obesity vs 34.8±2.5% with n =  total of 23 islets from 5 T2D patients). Differences in average expression were analyzed using unpaired two tailed Student's *t* test, and p values were represented on the graphs.

Recently, Butler and colleagues reported that nuclear localization of MAFA was abolished in beta cells of type 2 diabetes patients [17]. We analysed MAFA expression in pancreatic sections from 9 control nonobese, nondiabetic individuals, 4 obese non-diabetic individuals and 7 type 2 diabetic (T2D) patients. We found that MAFA expression was unaltered in islets from subjects with obesity as compared to normal individuals. On the contrary, among tested T2D patients, MAFA expression was completely lost in 2 patients, and shifted to a predominant cytoplasmic localisation in 3 patients (Figure 4A and B). More importantly, this deregulated MAFA nuclear expression was not only detected in beta cells, consistent with what has been reported by Butler *et al* [17], but also concurrently observed in alpha cells in the same samples. It was noticed that islet MAFA expression was not altered in the remaining two T2D patients, suggesting heterogeneity in molecular pathway alterations among the patients. We did not detect any significant change in PAX4 nuclear expression in alpha or beta cell from T2D patients, when compared with the subjects with obesity or control individuals (Figure 5A). However when analyzing the overall distribution of PAX4 expression, we observed a statistically significant decrease in the percentage of PAX4^+^ alpha cells per islet from the T2D cohort compared to individuals with obesity (respectively 37.2±3.7% vs 52.8±4.8%) (Figure 5B), whereas there was no significant change in the percentage of beta cells expressing PAX4 per islet in the same patients (Figure 5C).

## Discussion

Our present study revealed significantly different PAX4 and MAFA protein expression patterns in human islets compared to what was previously established in rodents. For the first time, our data uncover the expression of PAX4 and MAFA in approximately half of human adult alpha cells, while the expression of both factors is known to be exclusively present in beta cells during adult life in the mouse. Consequently, in humans, one might conclude that PAX4 and MAFA proteins are apparently neither specific markers nor specific transcriptional factors in beta cells.

It is noted that our data are consistent with the previous results from transcriptomic analysis of purified human islets, which indicate that PAX4 and MAFA expression at low transcription levels was indeed perceptible in alpha cell population [9]. The weak expression of both transcription factors in alpha cells and the lack of specific and sensitive tools for protein expression detection may explain at least partially the failure to detect them so far in human alpha cells. The current work confirmed the recent publication by Dai *et al* [10], who used a different anti-human MAFB antibody. Furthermore, by examining nuclear co-localization of PDX1/MAFB, we showed that MAFB is expressed in a fairly large population of PDX1-expressing cells. The discrepancy between the MAFB positivity in insulin^+^ cells found in our work (42% being the average of percentages of INS^+^MAFB^+^ and PDX1^+^MAFB^+^ staining combined) and that (9%) reported by Dai *et al*. [10] may mainly result from detection and counting methods. In particular, co-nuclear IF immunostaining used in the current study may facilitate a more accurate evaluation. How MAFB works and interacts with other transcriptional factors, MAFA in particular, within beta cells to exert its biological functions remains to be shown.

The data obtained from our study provide compelling evidence demonstrating the existence of subpopulations, in terms of protein expression, among human alpha and beta cells. It would, therefore, be relevant to investigate whether GLU^+^PAX4^+^ cells represent a functionally, and even developmentally, different population than the GLU^+^PAX4^−^ cells that are similar to alpha cells in rodent islets. Similarly, the same question could be applied to GLU^+^MAFA^+^ cells versus GLU^+^MAFA^−^ cells. It would be worth mentioning that, a recent study in rat islets indicated that functionally distinct beta cell sub-populations do exist [18]. Our data, therefore, may provide further molecular basis supporting the presence of these subpopulations and unknown cell plasticity in both alpha and beta cells of the adult human endocrine pancreas. The current finding may also urge further investigations on the capacity of these distinct subpopulations to transdifferentiate into insulin-secreting cells, a phenomenon recently discovered in rodents [15], [19]. To this end, it would be critical in the future, material permitting, to isolate and characterize these subpopulations.

The expression pattern of PAX4 and MAFA in human islet cells described in the current study urges one to reconsider their biological roles, as well as the possible impact of such distinct PAX4 and MAFA expression pattern in human pathology, since their deregulation could be involved in cell plasticity change in the diseases affecting islets, as suggested by the recent works [20]. Further studying PAX4, MAFA and MAFB expression, as well as their activities in human islet cells in different pathophysiological contexts, may provide useful clues to address these issues in the future. Lastly this work adds to a growing number of articles enforcing the imperative nature of research on human pancreatic islet cells.
